# Single-cell RNA sequencing of *Trypanosoma brucei* from tsetse salivary glands unveils metacyclogenesis and identifies potential transmission blocking antigens

**DOI:** 10.1073/pnas.1914423117

**Published:** 2020-01-21

**Authors:** Aurélien Vigneron, Michelle B. O’Neill, Brian L. Weiss, Amy F. Savage, Olivia C. Campbell, Shaden Kamhawi, Jesus G. Valenzuela, Serap Aksoy

**Affiliations:** ^a^Department of Epidemiology of Microbial Diseases, Yale School of Public Health, Yale University, New Haven, CT 06520;; ^b^Vector Molecular Biology Section, Laboratory of Malaria and Vector Research, National Institute of Allergy and Infectious Diseases, National Institutes of Health, Rockville, MD 20852

**Keywords:** African trypanosome, vaccine, single-cell RNA-seq, tsetse, metacyclic

## Abstract

Tsetse-transmitted African trypanosomes must develop into mammalian-infectious metacyclic cells in the fly’s salivary glands (SGs) before transmission to a new host. The molecular mechanisms that underlie this developmental process, known as metacyclogenesis, are poorly understood. Blocking the few metacyclic parasites deposited in saliva from further development in the mammal could prevent disease. To obtain an in-depth perspective of metacyclogenesis, we performed single-cell RNA sequencing (scRNA-seq) from a pool of 2,045 parasites collected from infected tsetse SGs. Our data revealed three major cell clusters that represent the epimastigote, and pre- and mature metacyclic trypanosome developmental stages. Individual cell level data also confirm that the metacyclic pool is diverse, and that each parasite expresses only one of the unique metacyclic variant surface glycoprotein (mVSG) coat protein transcripts identified. Further clustering of cells revealed a dynamic transcriptomic and metabolic landscape reflective of a developmental program leading to infectious metacyclic forms preadapted to survive in the mammalian host environment. We describe the expression profile of proteins that regulate gene expression and that potentially play a role in metacyclogenesis. We also report on a family of nonvariant surface proteins (Fam10) and demonstrate surface localization of one member (named SGM1.7) on mature metacyclic parasites. Vaccination of mice with recombinant SGM1.7 reduced parasitemia early in the infection. Future studies are warranted to investigate Fam10 family proteins as potential trypanosome transmission blocking vaccine antigens. Our experimental approach is translationally relevant for developing strategies to prevent other insect saliva-transmitted parasites from infecting and causing disease in mammalian hosts.

## Significance

African trypanosomes, *Trypanosoma brucei* spp., are transmitted by the bite of infected tsetse flies. Mammalian vaccines are not available, and diagnosis and treatment remain difficult in the affected remote areas. The transcriptomic analysis of individual parasites from infected tsetse salivary glands provides insight into the developmental processes that give rise to infective metacyclic parasites transmitted to the host bite site. We describe proteins associated with the different parasite developmental stages in salivary glands and specifically highlight a family of nonvariant surface proteins associated with metacyclic parasites. Immunization with one member of this family reduced parasitemia early in the infection in mice, promising to be a potential candidate antigen for a transmission blocking vaccine approach.

Many parasites of medical and agricultural significance rely on insect vectors for transmission. Among these are *Trypanosoma brucei* spp., the causative agent of trypanosomiasis across sub-Saharan Africa, which are transmitted to their mammal hosts via the saliva of tsetse flies during blood feeding (1–4). Over the course of their life cycle, the parasites undergo multiple developmental stages, reflective of changes that allow them to adapt and survive in the different environments they encounter in their vertebrate host and invertebrate vector. For trypanosomes, these changes include nutrient-specific metabolic fluctuations, structural modifications related to the cellular localization of the kinetoplast and nucleus structures, and the expression of unique glycosylphosphatidyl inositol (GPI)‐anchored surface coat proteins.

It has not been possible to develop effective mammalian vaccines to prevent trypanosomiasis. This is largely because, in the mammal, the parasites are covered with the predominant surface coat proteins, variant surface glycoprotein (VSG). The continuous turnover of the VSG coat, in addition to the sequential expression of antigenically unique VSG coat proteins, a process known as antigenic variation, enables trypanosomes to evade the vertebrate immune response and sustain an infection (5). Following ingestion by tsetse, the replicative bloodstream form of the parasites, known as slender cells, are lysed while insect-adapted and cell cycle-arrested stumpy cells differentiate to procyclic forms and acquire an invariant surface coat made up of procyclin proteins (6). To facilitate parasite midgut colonization, VSGs released into the midgut lumen by slender forms are taken up by tsetse’s cardia (also called proventriculus), where they transiently interfere with the production of a structurally robust peritrophic matrix (PM) midgut barrier (7). Following midgut colonization, procyclic parasites migrate to the cardia and foregut where they transform to long- and short-epimastigote forms (8). The short epimastigotes acquire yet another surface coat made up of *brucei* alanine-rich proteins (BARPs), colonize the SGs (9), and give rise to epimastigotes that undergo asymmetric division to give rise to premetacyclic cells (10). The premetacyclic cells acquire a different coat selected from ∼20 to 30 VSGs, termed metacyclic VSG (mVSG) (11, 12). The acquisition of the mVSG coat is accompanied by morphological changes, including rounding up of the posterior end, elongation of the flagellum, and repositioning of the kinetoplast to the posterior end (10, 13). The metacyclic forms are quiescent, nondividing, and arrested in G1/G0 (14). Finally, an antigenically heterogeneous population of mammalian infective-metacyclic trypanosomes, with each individual cell expressing a single mVSG, are released into the SG lumen (15–17) and deposited at the bite site via the saliva of blood-feeding tsetse flies.

While extensive knowledge on the interactions between bloodstream-form parasites and their mammalian host exists, information on the in vivo tsetse-specific trypanosome stages is sparse. High-throughput RNA sequencing (RNA-seq) analysis from the midgut, cardia, and SG tissues of parasitized tsetse flies helped profile *T. b. brucei* transcripts from different developmental stages (18). However, as multiple developmental forms of the parasite reside within each organ, particularly in SGs where parasites undergo maturation to infective cells, these approaches could not provide sufficient resolution to identify development-specific processes. A better understanding of mechanisms that give rise to mammalian infective metacyclic parasites, known as metacyclogenesis, is fundamental and can help with the development of new methods to interfere with disease transmission success.

In this study, we applied single-cell RNA sequencing (scRNA-seq) to profile the transcriptomic landscape from a pool of 2,045 individual *T. b. brucei* isolated from SGs, which include multiple developmental forms (epimastigote and pre- and mature stages of metacyclic forms). We mined our data for stage-specific transcripts and identified metabolic profiles that reflect the process of preadaptation to the mammalian nutritional environment. We also present immunological and cellular microscopy data on one protein localized to the surface of mature metacyclic cells. We provide preliminary data that support the utility of this protein as a potential candidate transmission blocking antigen.

## Results

### scRNA-Seq Reveals Three Distinct Clusters.

Multiple trypanosome developmental stages reside within infected tsetse SGs, ranging from proliferating epimastigotes to infective metacyclic forms adapted to survive in the mammalian host. We aimed to elucidate the molecular process of metacyclogenesis by characterizing the transcriptomic profiles of 2,045 individual parasites isolated from infected SGs that harbor a mix of different developmental stages. We obtained a total of 188,869,749 reads, 69% of which originated from identified cells (Dataset S1). We observed an average of 92,356 reads per cell and detected transcripts from 8,698 different genes across the profiled cells, with a median of 298 expressed genes detected per cell. Our gene expression analysis was based on unique molecular identifier (UMI) counts, with an UMI count corresponding to a unique transcript molecule. We observed a median of 410 UMI counts per cell.

**Fig. 1.**
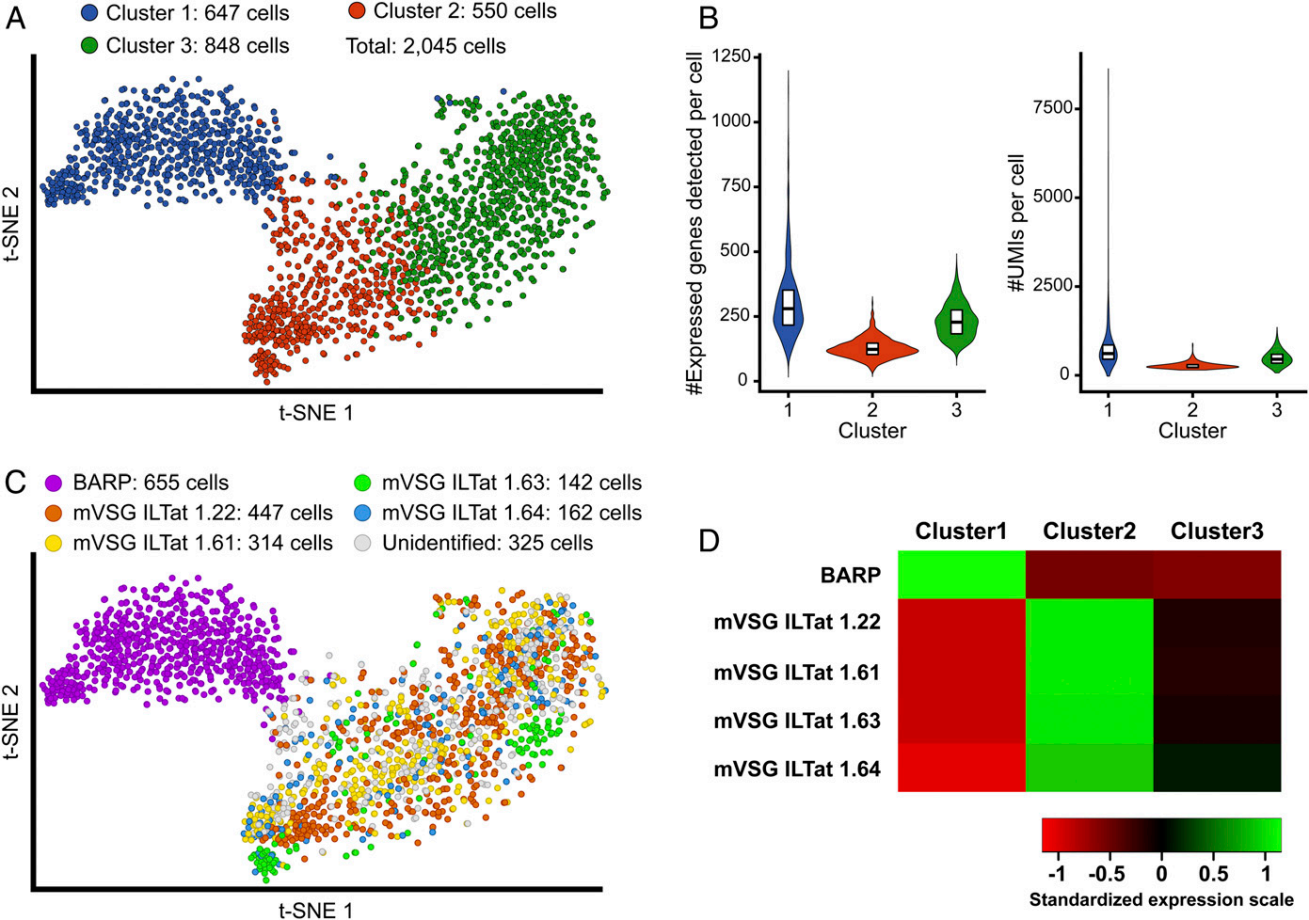
Clustering of SG-associated parasites based on scRNA-seq profiles. Clusters were determined using the Euclidean metric after determining the optimal number of clusters using the K-means method. Parasite cells were grouped in three clusters (K = 3) including 647 cells (cluster 1, blue), 550 cells (cluster 2, red), and 848 cells (cluster 3, green). (*A*) T-distributed stochastic neighbor embedding (t-SNE) plot of the 2,045 single-sequenced parasite cells. Colors indicate associated clusters. (*B*) Violin plots of the number of expressed genes detected per cell for each cluster (*Left*). Violin plots of the number of UMI counts per cell for each cluster (*Right*). Boxes represent the median and the first and third quartiles. (*C*) t-SNE plot of the 2,045 single parasite cells. Color indicates the main coat protein expressed by a given cell. Sequencing data for each individual BARP-coding gene were merged to represent the sequencing of one global coding gene: 655, 447, 314, 142, and 162 cells expressing BARP (pink), VSG ILTat 1.22 (orange), VSG ILTat 1.61 (yellow), VSG ILTat 1.63 (green), and VSG ILTat 1.64 (blue) as their main coat protein, respectively. A total of 325 cells were expressing neither BARP nor an identified VSG (gray). (*D*) Heatmaps of the averaged and standardized LSM expression value of the genes coding for the coat proteins in the three parasite cell clusters.

After normalization and exclusion of genes expressed in less than 5% of the cells, our data segregated into three major clusters comprised of 647, 550, and 848 cells based on the calculation of the Davies–Bouldin index (Fig. 1*A* and *SI Appendix*, Fig. S1). While cluster 1 was distinct, clusters 2 and 3 represented subdivisions of a broader continuous cluster. The number of genes detected expressed per cell in cluster 1 ranged between 91 and 1,126. Comparatively, we detected between 43 and 267 and 107 and 448 genes expressed from cells in clusters 2 and 3, respectively (Fig. 1*B*). Similarly, cluster 1 showed high variability in the number of UMI counts per cell, ranging from 184 to 8,402. Comparatively, UMI counts per cell in clusters 2 and 3 ranged from 183 to 844 and 186 to 1,210, respectively (Fig. 1*B*). For both measures, cluster 2 presented less variability concentrated around the median, while cluster 3 presented a broader distribution of data similar to cluster 1 but without the extreme variabilities. Altogether, these results indicate that parasites within clusters 1 and 3 are transcriptionally more active and diversified than those within cluster 2. Because gene expression is largely posttranscriptionally regulated in trypanosomes, we analyzed the translational activity associated with cells in different clusters by profiling the expression of 60S and 40S ribosomal RNA protein coding genes (*SI Appendix*, Fig. S2). Cluster 3 had the highest level of rRNA gene expression, indicative of greater translational activity associated with this cluster.

We next analyzed the expression of *barp* and *mVSG*s, which encode the surface coat proteins of epimastigote and metacyclic forms, respectively (19). We observed that a total of 655 cells, almost exclusively within cluster 1, expressed *barp* (Fig. 1*C*). The remaining cells in clusters 2 and 3 expressed one of the four mVSGs previously identified from parasitized SGs (18): ILTat 1.22, 1.61, 1.63, and 1.64 (Fig. 1*C*). This finding supports the previous observations that each metacyclic parasite presents a unique mVSG protein coat and that the metacyclic population transmitted to the mammal in saliva represents a heterogenous mix of cells, each with a different surface coat antigen (20, 21). The predominant *mVSG*s expressed in clusters 1 and 3 were ILTat 1.22 and 1.61 detected in 447 and 314 cells, respectively. The next most abundant mVSGs were ILTat 1.64 and 1.63 detected in 162 and 142 cells, respectively. When we compared the expression of coat proteins at the cluster level, instead of at the individual cell level, again *barp* was predominant in cluster 1, while mVSGs showed highest expression in cluster 2, with less expression in cluster 3 and none in cluster 1 (Fig. 1*D*). Interestingly, we also noted that 325 cells (16% of the total cells we analyzed) expressed neither *barp* nor one of the four known *mVSG*s. Because these cells are distributed within clusters 2 and 3, we speculate that they may encode other mVSGs that remain to be classified in the parasite strain we used here. We also conclude that *barp* and *mVSG*s, which are the most differentially expressed (DE) genes between cells in cluster 1 and clusters 2 and 3 (Dataset S1), serve as relevant biomarkers during development.

Altogether, our results indicate that based on transcriptional activity profiles and the identity of the surface coat proteins expressed, cluster 1 includes proliferative epimastigote stages (called cluster Epim), while clusters 2 and 3 represent different stages of metacyclics (called clusters Meta1 and Meta2, respectively) (22). Our scRNA-seq data indicate differences within the two metacyclic populations, with the rRNA profile indicating less translational activity in cells within Meta1 (*SI Appendix*, Fig. S2). In addition, cells within the Meta1 cluster had fewer UMI counts and fewer genes detected relative to Meta2 (*SI Appendix*, Fig. S3), suggesting that Meta1 is transcriptionally more quiescent than Meta2, which exhibits a more diverse transcriptome and higher transcriptional activity. This profile could reflect a process of transition within the metacyclic population, toward parasites that are ready to survive in the different nutritional and immune environment of the mammalian host.

### Predicted Metabolome of Parasites in Different Clusters Reflects a Process of Adaptation to Different Host Environments.

**Fig. 2.**
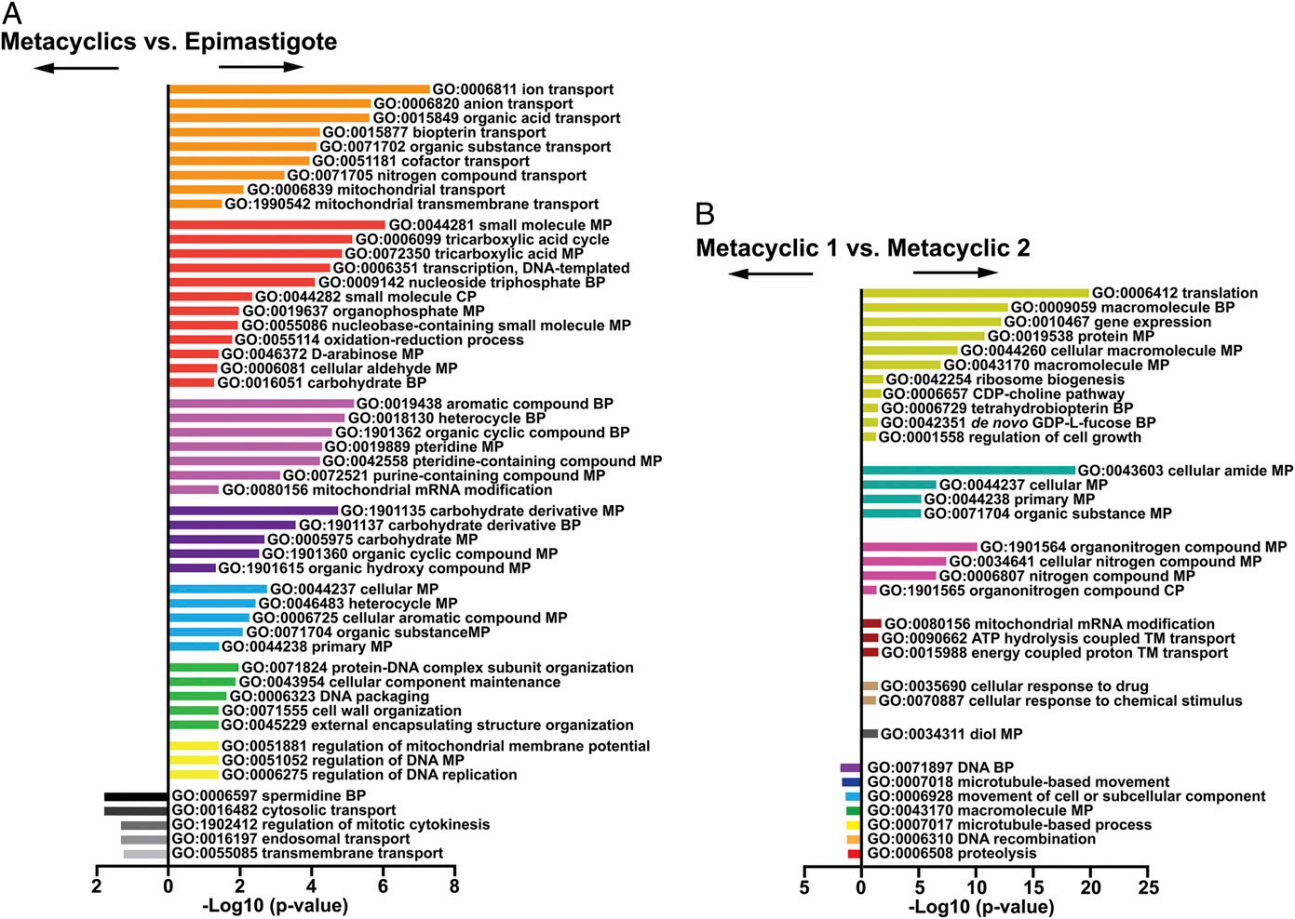
GO enrichment analysis of epimastigote and metacylic forms. (*A*) Biological process-associated GO terms significantly enriched in epimastigote forms (*Right* bars) in comparison to metacyclic forms (*Left* bars). (*B*) Biological process-associated GO terms significantly enriched in cells in Meta1 cluster (*Right* bars) in comparison to parasites in Meta2 (*Left* bars). In both comparisons, the GO terms have been filtered using the REVIGO webtool to avoid redundancies. Bars represent the level of significance of the term enrichment. Bars of the same colors represent terms that are closely associated.

To better understand the biological differences between the epimastigote and metacyclic parasites, we identified DE genes between cluster Epim and the combined clusters Meta1 and Meta2 (Dataset S1). We then applied a gene ontology (GO) enrichment analysis and summarized the redundant terms using the REVIGO webtool (23) for functional differences (Dataset S2 and Fig. 2*A*). GO terms enriched in Epim were preferentially associated with energy metabolism, such as mitochondria-associated anion transport and tricarboxylic acid (TCA) cycle reflective of oxidative phosphorylation, which is the major path for energy production in insect stage trypanosomes (24). By contrast, metacyclic form clusters were enriched in GO terms associated with cytosolic and transmembrane transport, amine biosynthesis, and spermidine biosynthesis, as well as regulation of cytokinesis (Fig. 2*A*). Spermidine biosynthesis is particularly relevant as spermidine is a precursor of trypanothione (25), a derivative of gluthathione that acts as an antioxidant necessary for trypanosomes to infect their vertebrate hosts (26). However, these results would need further functional validation due to a single gene associated with the GO term “spermidine biosynthesis.” Altogether, these results support the expected metabolic profile for epimastigote forms that utilize mitochondria and an amino acid-based metabolism. In contrast, the putative metabolome of metacyclic forms reflects a process of adaptation to the mammalian glucose-rich nutritional environment where the bloodstream-form parasites depend upon glycolysis for ATP production (22, 27).

We observed that while epimastigote cells clustered tightly, metacyclic trypanosomes were distributed over two clusters. To further investigate the differences between the two metacyclic clusters, we identified DE genes between Meta1 and Meta2. Similar GO enrichment analyses revealed that Meta1 is enriched in functions associated with DNA biosynthesis and microtubule-based movement, while Meta2 is enriched in functions associated with gene expression and translation, including for CDP-choline pathway, nitrogen-based metabolism, and general biosynthesis pathways (Dataset S2 and Fig. 2*B*). We hypothesized that these metabolic differences, along with our earlier observations showing greater translational activity in Meta2, indicate that Meta1 parasites begin the reorganization of cellular structures, while Meta2 parasites are transitioning to a metabolically more active state as precursors of bloodstream forms in the mammalian host.

### Subcluster-Specific Expression Profiles.

**Fig. 3.**
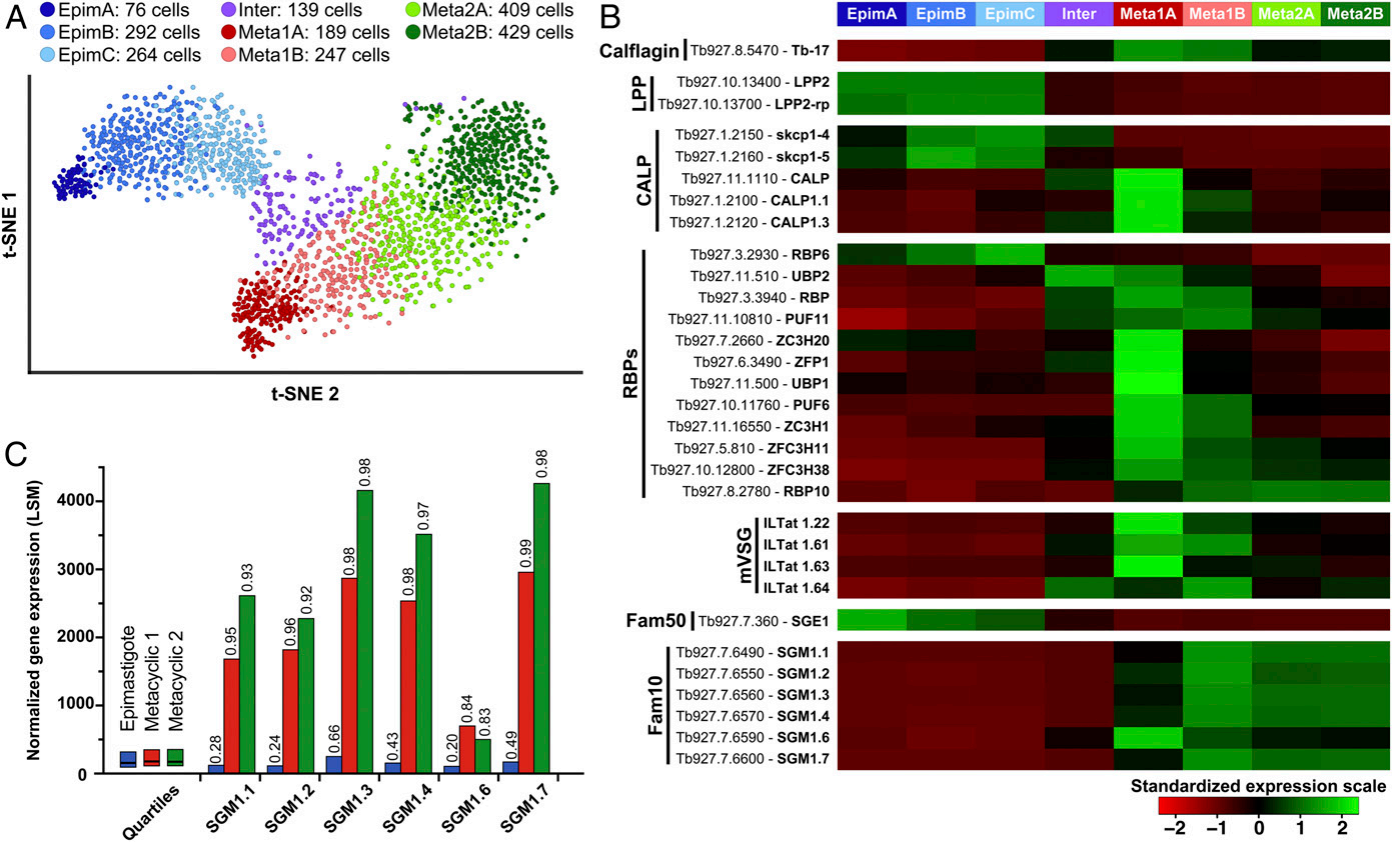
Cluster-specific gene expression profiles. Parasite cells within the clusters Epim, Meta1, and Meta2 were further divided into eight subclusters. Epim includes 76, 292, and 277 cells clustered as EpimA (dark blue), EpimB (blue), and EpimC (light blue), respectively. Meta1 includes 189 and 223 cells clustered as Meta1A (dark red) and Meta1B (light red), respectively. Meta2 includes 433 and 433 cells clustered as Meta2A (dark green) and Meta2B (light green), respectively. A total of 122 cells located at the intersection of clusters Epim, Meta1, and Meta2 were clustered as an intermediary cluster (Inter, violet). (*A*) t-SNE plot of the 2,045 single-sequenced parasite cells. Colors indicate associated cluster. (*B*) Heatmaps of the averaged and standardized LSM expression value of genes encoding LPPs, CALPs, RBPs, the four prominent mVSGs, SGE1 proteins in Fam50, and SGM1 proteins in Fam10 in the eight subclusters. Sequencing data for the five individual SGE-coding gene were merged to represent the sequencing of one global coding gene. ZC3Hx, zinc-finger proteins; PUF, pumilio family RBP; SGM, salivary gland metacyclic protein. (*C*) LSM of the Fam10 member gene expression values in clusters Epim (blue), Meta1 (red), and Meta2 (green). For each cluster, the first, second (median), and third quartiles are represented as a point of comparison. Percentiles of genes that are less expressed than the represented gene are given on top of the bars.

To dissect the process of differentiation into mature metacylics, we further split the original three clusters into seven subclusters: three within Epim, two in Meta1, and two Meta2 (Fig. 3*A*). We manually formed an eighth subcluster (called Inter for intermediary subcluster) that contains the cells lying at the junctions of the original three clusters. These subclusters reflected a finer scale of varying gene expression capacity, based on the number of genes expressed and UMI counts, than those described for the original three clusters (*SI Appendix*, Fig. S3). We next investigated the spatial expression profile of putative proteins known to regulate developmental processes in African trypanosomes within the eight subclusters. We focused on DE genes expressed in at least one of the three original clusters based on pairwise comparisons (Dataset S1 and Fig. 3*B*).

Prior studies had identified two putative lipid phosphate phosphatases (LPPs) expressed in SG stages (28), which our data confirmed to be epimastigote specific. We also investigated flagellar calcium-binding proteins, calflagins, which have been associated with premetacyclic and metacyclic forms, but not with epimastigote parasites (10). Our data confirmed the preferential expression of *calflagin* in the Meta1A–Meta1B cluster, which further supports that these clusters represent the premetacyclic form. We next evaluated expression profile of calpains, which enable the development and interaction of the related parasite *Trypanosoma cruzi* with the kissing bug vector’s midgut cells (29, 30). We noted high expression of calpain-related (*skcp1-4* and *skcp1-5*) genes in epimastigote forms, while calpain genes (*calp*, *calp1.1*, and *calp1.3*) were abundantly expressed in Inter, Meta1A, and Meta1B clusters, with the highest expression noted in the subcluster Meta1A. Hence, changes in the expression of calpains could be one factor that regulates tsetse–parasite interactions in SGs during metacyclogenesis.

African trypanosomes produce polycistronic immature mRNAs, which are processed to mature monocistronic mRNAs by transsplicing and polyadenylation events. Essential components of this process are structural motifs enriched in 3′-untranslated regions of mRNAs that serve as ligands for different RNA binding proteins (RBPs), which inhibit the translation of their target mRNAs (31, 32). Proteins that regulate these processes, including members of RBPs, zinc-finger (ZF), pumilio (PUF), and U-rich RNA binding protein (UBP) families, presented a varying profile with the highest number expressed in Meta1, including the zinc-finger domain-containing proteins (ZC3H20, ZFP1, ZC3H1, ZFC3H11, and ZFCH38) and UBP1 (Fig. 3*B*). Moreover, both the number of genes expressed and UMI counts in Meta1A were lower than in Meta2B (*SI Appendix*, Fig. S3), indicating that Meta1A is the least transcriptionally active group of cells as we proposed earlier. We noted that RBP6 was predominant in EpimC, which represents cells transitioning to metacyclic forms. In fact, RBP6 overexpression in cultured procyclic forms resulted in differentiation to long and short epimastigotes as well as mammalian infective metacyclic parasites, supporting the role of RBP6 in parasite development regulation (33). We also observed the highest expression of *rpb10* in the Meta2 population, which are preparing to transition to proliferative bloodstream forms in the mammal. Similar overexpression of RBP10 in procyclic forms increased the abundance of bloodstream form-specific transcripts (34) and initiated the transition of cells to the bloodstream stages (35, 36). High levels of *rbp10* expression were also noted in an in vitro metacyclic cell line generated by RBP6 overexpression in comparison to procyclic cells (22). Finally, the mVSG genes were expressed abundantly within the Meta1 subclusters, signifying the transition of the epimastigote state cells to the premetacyclic forms at this time.

Taken together, our results suggest that trypanosomes undergo three sequential developmental steps in tsetse’s SGs, each with varying transcriptomic and metabolomic profiles. Specifically, epimastigote forms (Epim cluster) transition to premetacyclic forms (Meta1 cluster), which give rise to mature metacyclic forms (Meta2 cluster). We hypothesize that mature metacyclic forms populating cluster Meta2 represent the free mammalian infective population transmitted to the bite site in fly saliva.

### Unique Surface Proteins Associated with Epimastigote and Metacyclic Forms.

**Fig. 4.**
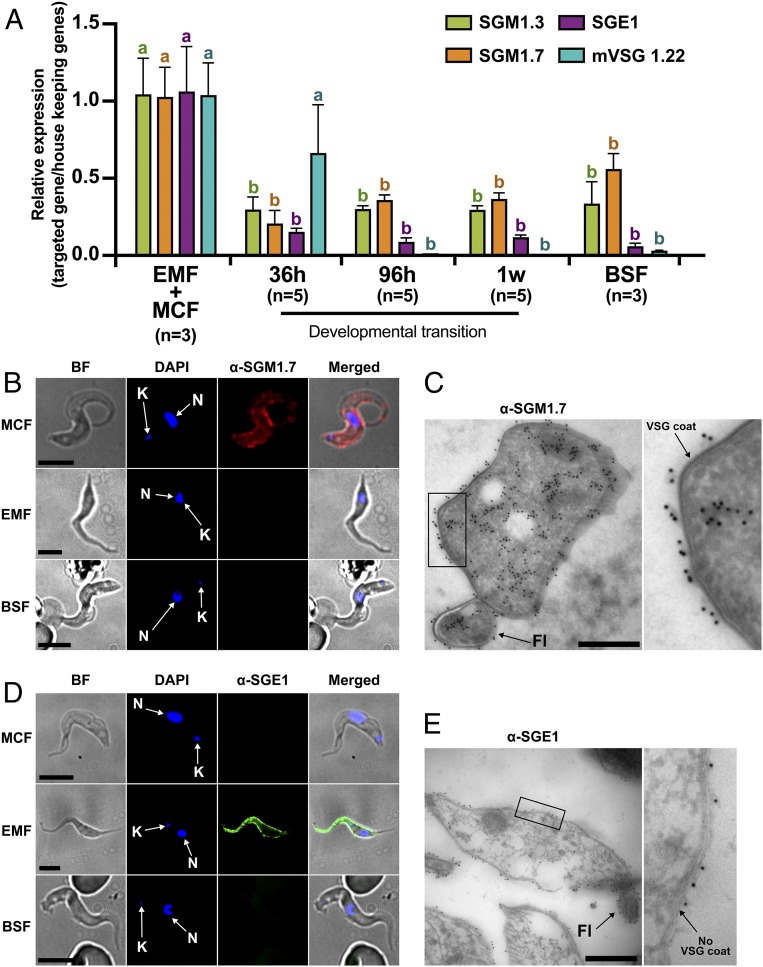
Expression and cellular localization of SGM1.7 and SGE1. (*A*) qRT-PCR analysis of *sgm1.3*, *sgm1.7*, *sge1*, and *mVSG1.22* expression in infected SG and in mice blood purified temporally postchallenge with SG purified parasites. Data were normalized using the geometrical mean of the expression of DNA ligase I, AATP5, and PM20. EMF, epimastigote form; MCF, metacyclic form; BSF, bloodstream form. Statistical analysis was conducted using a one-way ANOVA followed by Tukey’s HSD post hoc test for pairwise comparisons. For each gene, statistical significance is represented by letters above each condition, with different letters indicating distinct statistical groups (*P* < 0.05). Complete statistical results are available in Dataset S4. (*B*) Immunofluorescence assay of SGM1.7. Red, anti-rSGM1.7; blue, DAPI. BF, bright field; N, nucleus; K, kinetoplast. (Scale bars: 5 µm.) (*C*) Immunogold staining for localization of SGM1.7. Black dots represent 10 nm colloidal gold-labeled protein A binding to antibodies specific for SGM1.7. *Right* is a magnification of the *Left* black frame. Fl, flagellum. (Scale bar: 500 nm.) (*D*) Immunofluorescence assay of SGE1. Red, anti-rSGE1; blue, DAPI. N, nucleus; K, kinetoplast. (Scale bars: 5 µm.) (*E*) Immunogold staining for localization of SGE1. Black dots represent 10 nm colloidal gold particles labeled protein A binding to antibodies specific for SGE1. *Right* is a magnification of the *Left* black frame. Fl, flagellum. (Scale bar: 500 nm.)

We searched within the different clusters for abundant and differentially expressed genes that encode surface associated proteins. The first family we noted, here named salivary gland epimastigote 1 (SGE1), includes five closely related members exclusively expressed in clusters EpimA–C, (Fig. 4*A* and *SI Appendix*, Fig. S4). Previous in silico analysis identified the SGE1 family as putative cell surface proteins of African trypanosomes (Fam50, lineage *iv*) (37). Expression profiling in different tsetse tissues indicated its preferential expression by SG parasites (38). Further, SGE1 proteins were not detected within the surface phylome of bloodstream forms when analyzed by biochemical methods (39).

We identified a second gene family (family 10, Fam10), comprised of six closely related genes, expressed at high levels exclusively in Meta1 to Meta2 subclusters (37) (Fig. 3*A* and *SI Appendix*, Fig. S5) (18). Least-square means (LSM) expression values for the Fam10 genes were highest in cluster Meta2 (with the exception of *sgm1.6*, Fig. 3*C*). The peak expression of Fam10 genes was within the subcluster Meta1B (Fig. 3*B*). This expression pattern could be indicative of their preferential function during transition from pre- to mature metacyclic forms.

To understand the temporal processes during differentiation from metacyclic to bloodstream-form parasites in the mammal, we measured the transcript levels for stage-specific genes (Dataset S3 and Fig. 4*A*). Expression of *mVSG1.22*, which is one of the most abundant genes expressed in our metacyclic parasite line in the SG organ, persisted in the mammalian host (mouse blood) for the first 36 h, but was undetectable thereafter as cells differentiated to bloodstream forms (Fig. 4*A*). Transcripts for *sgm1.3* and *sgm1.7*, which are two members of the Fam10 family most abundantly expressed in metacyclic cells (Fig. 3*C*), continued to be detected in the bloodstream-form cells in the mammalian host, albeit at significantly lower levels compared to the metacyclic stage (Fig. 4*A*). In accordance, a biochemical analysis of parasite surface proteome identified both SGM1.3 and SGM1.7 in the bloodstream-form trypanosomes (39). Similar profiling of epimastigote-specific SGE1 (here analyzed one member, *Tb927.7.360*) showed high expression in the SG organ followed by a sharp decline in the mammalian host by 36 h and beyond (Fig. 4*A*). Collectively, it appears that metacyclic cells persist in the mammalian host for about 36 h before transforming into the bloodstream-form cells, and that SGM1.7 and SGM1.3 proteins, most abundant in metacyclic forms, continue to be expressed in the bloodstream forms, albeit at significantly lower levels.

We next evaluated the cellular localization of SGM1.7 and SGE1 using immunofluorescence and immunogold microscopy analyses. In support of our transcript data, SGM1.7 is localized specifically to the surface of metacyclic cells (Fig. 4 *B* and *C*) while SGE1 is detected only on the surface of epimastigote forms (Fig. 4 *D* and *E*). Interestingly, while we detected *sgm1.7* expression in the bloodstream forms, we could not establish its presence on the surface of cells by immunofluorescence. It may be due to the thick VSG coat of the bloodstream form that prevents detection of nonvariable surface antigens. It is also possible that the abundance of the SGM1.7 protein may vary between the metacyclic and bloodstream-form parasites, independent of the *sgm1.7* transcript levels associated with each developmental state.

### SGM1.7 Explored as a Transmission Blocking Vaccine Antigen.

Because SGM1.7 is exposed on the surface of infective metacyclic parasites, we explored whether SGM1.7 could be a potential target for transmission blocking studies. We used SGE1 as a control since this protein is exclusively expressed in the epimastigote forms that are not infectious or free in saliva to be introduced into the mammalian host.

**Fig. 5.**
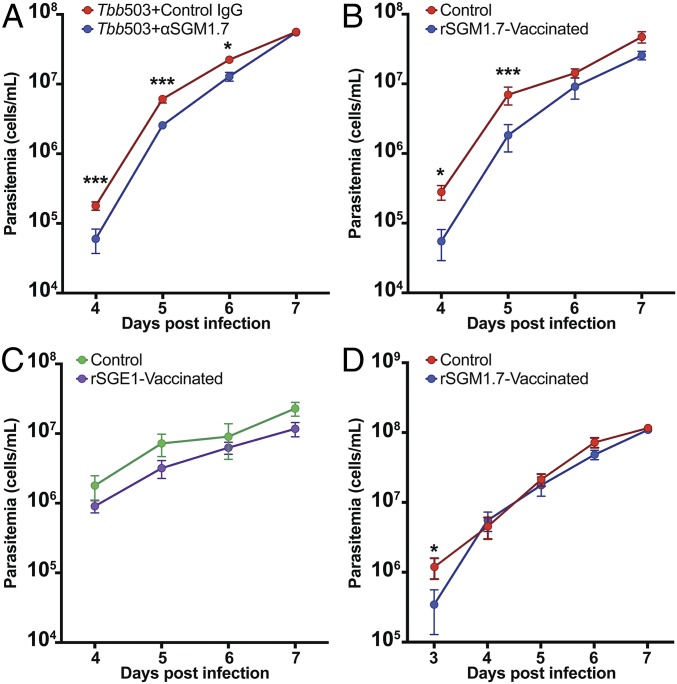
Prophylactic efficacy of SGM1.7. (*A*) Antimetacyclic-specific rSGM1.7 IgG and naive mice IgG coinoculated with 500 SG parasites intraperitoneally in two groups of mice, respectively. Parasitemia was followed via eye bleeds. (*B* and *C*) Two independent groups of mice vaccinated with metacyclic-specific rSGM1.7 or epimastigote-specific rSGE1 were challenged with 500 SG parasites injected i.d. in the ear. As a control, nonvaccinated age-matched groups of mice were similarly infected. Parasitemia was followed via eye bleeds. (*D*) Infections in mice vaccinated with metacyclic-specific rSGM1.7 or age-matched naive controls were initiated through a single infected fly bite intraperitoneally. For statistical analysis, a mixed-effect linear model was fitted, where the logarithm in base 10 of the parasitemia counts was taken as the dependent variable. For day-to-day pairwise comparison of parasitemia between vaccinated and nonvaccinated mice, significance was evaluated with Tukey’s contrasts. Statistically different data points are shown by asterisks: * > 0.05, *** > 0.0001. Complete statistical results are available in Dataset S4.

We first tested whether coinoculation of metacyclic parasites together with anti-SGM1.7 immunoglubulin G (IgG) could interfere with trypanosome development and block or reduce the onset of parasitemia in the mouse system. Using needle injection, we cointroduced intradermally 500 SG isolated parasites with anti-SGM1.7 or naive IgG, respectively. We followed the parasitemia in mice blood daily over the first week postchallenge and noted that presence of anti-SGM1.7 IgG reduced the parasitemia early in the infection process relative to control mice that received the same infectious parasites together with naive mouse IgG (Fig. 5*A*). This outcome indicates that an antibody-mediated response could interfere with parasite survival or differentiation at the bite site.

We next vaccinated mice with rSGM1.7 or rSGE1 in combination with adjuvant and confirmed the presence of high antibody titers (<1:312,500) in vaccinated animals (Dataset S5). We next challenged all vaccinated animals and age-matched naive mouse controls similarly with 500 parasites obtained from SGs. In two independent experiments, the parasitemia levels in rSGM1.7-vaccinated animals were lower than naive controls early in the infection (Fig. 5*B*). On the other hand, rSGE1-vaccinated animals did not show any difference in parasitemia levels relative to naive controls over the same time span analyzed (Fig. 5*C*). We also infected mice vaccinated with rSGM1.7 through a single infected tsetse bite and observed significantly lower parasitemia levels relative to naive controls at 3 d postinfection, the earliest time point measured (Fig. 5*D*). Although we did not observe sterile immunity via rSGM1.7 antigen, the significant decrease in the parasitemia measured early in vaccinated animals, suggests that some, but not all, metacyclic parasites were cleared from progressing to bloodstream infections at the bite site.

## Conclusions

The ability to capture the transcriptomes of individual parasites in infected tsetse SGs provides unmatched depth of insight into the developmental processes that give rise to mammalian infective metacyclic cells. Although posttranscriptional mechanisms that affect mRNA stability, translation, and protein stability regulate trypanosome gene expression, we observed temporal expression of several genes, which correlates well with the experimentally confirmed roles of their products during development, i.e., calflagins, calpains, RBP6, and RBP10. In addition, the predicted metabolomes of parasites in different clusters reflect a changing landscape from the known biochemical profile of the epimastigotes, which utilize mitochondria and an amino acid-based metabolism, to metacylic forms, which undergo development to survive in the glucose-rich environment of the mammalian host. Our data also confirm that each metacyclic cell expresses a single gene encoding one of the known mVSG surface coat proteins. In addition, within the pool of metacyclic cells that express mVSGs, we observed varying physiological states which likely reflect the process of gaining mammalian infectivity and adaptation to the mammalian host environment. Finally, our cell level data indicate that the metacyclic state represents a spectrum of cells with low but varying gene expression activity. Interestingly, the mature metacyclic parasites (Meta2 cluster) express higher numbers of genes, UMI counts, and rRNA transcripts relative to the premetacyclic state (Meta1 cluster). This heightened transcriptional state may promote the process of cellular development that takes place as the parasites entering into the mammalian bite site differentiate to bloodstream-form cells.

African trypanosomiasis continues to be a debilitating disease of great medical and socioeconomic importance. Because trypanosomes have evolved efficient mechanisms to manipulate and escape host immune responses, development of effective mammalian vaccines has not been possible (40). However, the ability to restrict infections at the bite site where relatively few parasites are inoculated can provide a powerful new method for disease prevention. In fact, experiments where mice were challenged twice with *Trypanosoma congolense*-infected tsetse, each challenge followed up by experimental cure, resulted in sterile immunity upon subsequent challenge (41). However, immunity to challenge was short-lived and did not provide long-term protection. It was thought that this loss of immunity could have resulted from the small number of parasites the animals typically received via fly challenge (42).

Building on the molecular knowledge of metacyclic infective forms, we identified a prominent nonvariant coat protein (SGM1.7) present on the surface of metacyclic cells. The putative role of Fam10 surface proteins in metacyclogenesis remains to be elucidated. Future experiments with genetically modified parasites can shed light on processes leading to survival in the SG, or during infection establishment in the mammalian host bite site. Our results with anti-SGM1.7 antibodies implicate a B cell antibody-mediated response that can interfere with parasite survival or differentiation during the initial stages of infection in the mammal. Although we did not observe sterile immunity in our experimental system, future experiments with SGM1.7 antigen can identify critical epitopes that are effective for parasite interference, thus improving the protein’s efficacy as a subunit vaccine antigen. In addition, including different members of the FAM10 family proteins, which are also highly expressed in metacyclic cells, together with SGM1.7 as vaccine antigens, could enhance immune protection. Given that Fam10 proteins are conserved in the *T. brucei* spp. parasites, including the human infective *T. b. rhodesiense* and *T. b. gambiense*, the availability of a transmission blocking vaccine can significantly enhance the disease prevention toolbox. Finally, tsetse saliva proteins have also been shown to influence the outcome of infections at the bite site by modulating host immune responses (43). Immunological strategies that target both saliva antigen functions and metacyclic-specific antigens can provide novel means to prevent disease progression early in the infection process when parasites are most vulnerable in the mammalian host.

## Materials and Methods

### Biological Material and Ethical Consideration.

Six- to 8-wk-old BalbC female mice were used for all of the vaccination experiments in strict accordance with the Yale University Institutional Animal Care and Use Committee (Protocol 2014–07266 renewed on May 2017). The insectary maintenance conditions for *Glossina morsitans morsitans* and *T. b. brucei* (RUMP 503) isolations in rats and infection establishment in flies are described in detail (*SI Appendix*, *Materials and Methods*).

### scRNA-Seq Library Preparation and Sequencing.

Parasites were obtained from SGs as described (*SI Appendix*, *Materials and Methods*). We aimed to recover a total of 3,000 cells for scRNA-seq library preparation. We used the Chromium Single-Cell 3′ Reagent Kits v2 Chemistry (10xGenomics) according to the manufacturer recommendation as described (*SI Appendix*, *Materials and Methods*), and paired-end sequencing was carried out at the Yale Center for Genome Analysis using the HiSeq2500 system (Illumina).

### scRNA-Seq Data Processing.

Mapping and counting were processed using the Cell Ranger software v2.1.1 (10xGenomics). Reads were mapped to *T. b. brucei* TREU927 (*Tbb*927) reference genome using the Cell Ranger *count* function as described (*SI Appendix*, *Materials and Methods*) and output files containing the single cell counts were transferred to the Partek Flow software (Partek Inc). The analyses for DE genes were generated using Partek Flow gene-specific analysis (GSA). For these analyses, gene expression values were assessed by the LSM of normalized UMI counts per cluster. To produce the heatmaps, LSM values have been adjusted to follow the standard normal distribution. Heatmaps were generated using R 3.5.1 (44).

### GO Analysis.

GO terms enriched within the DE genes between different developmental stages were determined by Fisher’s exact test using the GO enrichment tool on the TriTrypDB webserver (https://tritrypdb.org/tritrypdb/) (45) and summarized by removing redundant terms using the REVIGO web-based software (23) and setting up the allowed similarity at 0.5 (smaller list of GO terms).

### Differential Gene Expression Analysis.

For qRT-PCR analysis, three biological replicates were obtained from bloodstream trypanosomes purified from rats and from multiple pools of infected SGs. RNA was also analyzed from three groups of mice blood, each with five animals, 36 h, 96 h, and 1 wk postneedle inoculation with 4 × 10^4^ SG purified parasites. Trypanosome gene-specific primer sequences and amplification conditions are described in *SI Appendix*, Table S1. Statistical significance was determined by a one-way ANOVA followed by Tukey’s honestly significant difference (HSD) post hoc test, using Prism 8.2 (GraphPad).

### Recombinant Protein Expression.

For epimastigote-specific rSGE1 (Tb927.7.360, referred to here as SGE1) and metacyclic-specific rSGM1.7 (Tb927.7.6600, referred to here as SGM1.7) production, mature protein coding regions were amplified without the signal peptide (*SI Appendix*, Table S1), cloned into pET-28a expression vector (Novagen), and recombinant protein (rProtein) was induced, purified, and analyzed for purity using standard methods (*SI Appendix*, *Materials and Methods*).

### Immunofluorescence Analysis.

For immunostaining, parasites obtained from infected SG were fixed in 4% paraformaldehyde (PFA) and 0.2% gluteraldehyde and then processed as described in *SI Appendix*, *Material and Methods*. Cells were not permeabilized in order to detect surface exposure of the antigens. Slides were incubated with either rabbit anti-rSGE1 or mouse anti-rSGM1.7 or their respective preimmune sera. Alexa Fluor 488-labeled goat anti-rabbit IgG (Invitrogen) and Alexa Fluor 594-labeled goat anti-mouse IgG were used as secondary antibodies. Slides were visualized at 400× and 1,000× using a Zeiss Axio Imager M2 microscope and images were captured using an AxioCam Mrm (Zeiss) and the AxioVision40 software (v4.8.2.0, Zeiss). Images were processed using the Fiji version of the ImageJ software (46). Only contrasts and luminosity were adjusted on images.

### Electron Microscopy.

Parasites were collected by incubating infected SGs in phosphate saline glucose (PSG) at room temperature for 45 min and processed as described in *SI Appendix*, *Material and Methods*. For SGM1.7, sections were incubated with either preimmune or anti-rSGM1.7 mouse sera, then with a rabbit anti-mouse IgG secondary antibody used as a bridge to improve binding with 10 nm colloidal gold-labeled protein A (University Medical Center Utrecht [UtrechtUMC]). For SGE1, sections were incubated with either preimmune or anti-rSGE1 rabbit sera, and then with 10 nm colloidal gold-labeled protein A (UtrechtUMC). In both cases, samples were processed for viewing with an FEI Tencai Biotwin transmission electron microscope at 80 kV. Images were taken using a Morada CCD camera and iTEM software (Olympus).

### Coinoculation of Parasites with Immune Sera.

Five hundred SG purified parasites were mixed with 20 μg rSGM1.7 IgG in a volume of 20 μL or with 20 μg naive mice IgG and introduced intradermally (i.d.) in the ear via needle injection, respectively. Parasitemia was followed by retroorbital sampling of blood on days 4 to 7 in two independent experiments (Exps) (Dataset S5, Exps 2 and 3.

### Parasite Challenge of Vaccinated Mice.

For each experiment, two groups of mice with a minimum of five individuals were vaccinated with purified rSGE1 and rSGM1.7 with Magic Mouse adjuvant (Creative Diagnostics, product no. CDN-A001), receiving two boosts in 2-wk intervals. ELISA was performed on all vaccinated animals 2 wk postfinal boost (Dataset S5, Exps 4, 6, 8, and 10) and 3 wk after the final boost using a BioTek Synergy HT plate reader. Subsequently, mice were infected with 500 SG isolated parasites (containing epimastigote, premetacyclic, and mature metacyclic forms) via needle injection in the ear. Parasitemia was followed in two separate experiments (Dataset S5, Exps 5 and 7 for rSGE1 and Exps 9 and 11 for rSGM1.7). Age-matched naive mice were similarly infected as controls. Mice vaccinated with rSGM1.7 and naive age-matched controls were also infected via a single tsetse bite and parasitemia similarly monitored (Dataset S5, Exps 12 and 13).

### Data Availability.

Sequencing read files have been deposited in the NCBI BioProject database (ID PRJNA562204).

## Supplementary Material

Supplementary File

Supplementary File

Supplementary File

Supplementary File

Supplementary File

Supplementary File
