# Supplementary material for: Single-cell RNA sequencing of Trypanosoma brucei from tsetse salivary glands unveils metacyclogenesis and identifies potential transmission blocking antigens
Source: Proc Natl Acad Sci U S A. 2020 Jan 21;117(5):2613–21. doi: 10.1073/pnas.1914423117 (PMC7007551; doi:10.1073/pnas.1914423117)
Supplement: Supplementary File [file pnas.1914423117.sapp.pdf]

Supplementary Information Appendix

Supplementary Information for:

"Single-cell RNAseq of *Trypanosoma brucei* isolates from tsetse salivary glands: metacyclogenesis and transmission blocking antigens"

Aurélien Vigneron, Michelle B. O'Neill, Brian L. Weiss, Amy F. Savage, Olivia C. Campbell, Shaden Kamhawi, Jesus G. Valenzuela and Serap Aksoy*

*Corresponding author
Email: serap.aksoy@yale.edu

This PDF file includes:

Supplementary Material and Methods
Tables S1
Figures S1 to S5
SI References

Other supplementary materials for this manuscript include the following:

Datasets S1 to S5

www.pnas.org/cgi/doi/10.1073/pnas.1914423117

Materials and Methods

Biological material and Ethical consideration. Six to eight week old BalbC female mice were used for all the experiments described here (purchased from Charles River). This work was carried out in strict accordance with the recommendations of the Office of Laboratory Animal Welfare at the National Institutes of Health and the Yale University Institutional Animal Care and Use Committee (IACUC). The experimental protocol was reviewed and approved by the Yale University IACUC (Protocol 2014-07266 renewed on May 2017).

The insectary maintenance conditions for *Glossina morsitans morsitans* were maintained in Yale's insectary at 24°C with 50-55% relative humidity. All flies received defibrinated bovine blood (Quad Five #910-1000, Montana) every 48 hours through an artificial membrane feeding system. Only female flies were used in this study. Bloodstream form *T. b. brucei* (RUMP 503) were expanded in rats. Flies were infected by supplementing the first blood meal of newly eclosed flies (teneral) with $5x10^6$ parasites/ml using an artificial membrane system (1). Cysteine (10µM) was added to the infective blood meal to increase the infection prevalence (2). After a single parasite challenge, flies were maintained on bovine blood provided every other day. At least 30 days post initial parasite feeding, flies were microscopically dissected 72h after the last blood meal and SG checked for the presence of parasite. For challenge experiments, positive SG were placed in PSG (PBS with 2% glucose) buffer and crushed by pipette-tip to break open the SG and allow the parasite to be released into the buffer. The parasite number was determined microscopically and diluted to allow for 100 parasites/1ul. 5µl or 500 parasites were used for intradermal (ID) injection.

scRNA-seq library preparation and sequencing. Ten pairs of infected SG, dissected as described above, were severed into pieces and placed in 100µl of PSG to allow the content of the lumen, including parasites, to flow out for 10 min at RT. The solution was collected and centrifuged for 5 min at 30g to pellet the SG, and the supernatant containing the trypanosomes was transferred to a fresh tube. Trypanosome density was microscopically evaluated using a hemocytometer and adjusted to 700 cells/µl prior to processing for scRNA-seq library preparation. The library preparation was processed using the Chromium Single Cell 3' Reagent Kits v2 Chemistry (10xGenomics, California) according to the manufacturer recommendation. For optimizing our library preparation with the aim to recover 3,000 cells, we followed the manufacturer's recommendations by using a volume of 7.5µl of suspended cells concentrated at 700 cells/µl. To identify mRNAs originating from the same cell, the procedure samples a pool of ~3,500,000 10x Barcodes to separately index the transcriptome from each cell. It does so by partitioning thousands of cells into nanoliter-scale Gel Beads-in-emulsion (GEMs), where all generated cDNAs share a common 10x Barcode. During the library preparation, each cDNA is barcoded specifically for their originating cell and each cDNA molecule gets a Unique

Molecular Identifier (UMI). Hence, each UMI corresponds to a unique transcript, for which the cellular origin is traceable. After library completion, paired-end sequencing was carried out at the Yale Center for Genome Analysis using the HiSeq2500 system (Illumina, California). Read files have been deposited in the NCBI BioProject database ID# PRJNA562204.

scRNA-seq data processing. For *Tbb* RUMP503 strain used in this analysis, the VSG encoding genes are not fully identified. Although the exact lineage information on RUMP 503 is not available, it is a derivative of EATRO 795 fly-transmissible ILTat 1.3 (http://tryps.rockefeller.edu/trypsru2_pedigrees.html). A previous study where we investigated *Tbb* RUMP503 gene expression from infected SG organ identified four mVSGs most similar to ILTat VSGs; ILTat 1.22, ILTat 1.61, ILTat 1.63 and ILTat 1.64 (3). We included these four mVSG coding sequences with the genome of *Tbb* strain TREU927/4 GUTat10.1 release 40 (www.tritrypdb.com) to generate a suitable reference genome for our analysis.

Prior to mapping the reads produced from sequencing analysis, a reference package was generated from our customized *Tbb* TREU927 genome using the *mkref* function of Cell Ranger software v2.1.1 (10xGenomics, Pleasanton, CA). Reads were then mapped to the created reference package using the Cell Ranger *count* function following the manufacturer pipeline that are summarized thereafter. Cell Ranger uses the STAR software for reads alignment. In the used pipeline, the software defines exonic reads when at least 50% of a read intersects an exon. Whenever an exonic read mapped to both a unique exonic locus and one or more non-exonic loci, the read was considered to be confidently mapped to the exonic locus. Only exonic reads mapping to a unique annotated transcript were considered confidently mapped to the transcriptome and retained for UMI counting. Each observed barcode, UMI and gene combination is recorded as a UMI count, which corresponds to a unique transcript originating from an identified cell.

Subsequently, Cell Ranger uses a proprietary algorithm to discriminate between barcodes that correspond to reliable cells and background (this latter including lower quality cells due to lower UMI counts and ambient RNA). The algorithm relies on an UMI-based cut-off to determine the barcodes that will be identified as reliable cells. That cut-off is determined based on the input data as follows. First, the UMI counts of all the identified barcodes are ranked in a decreasing order. The software then selects the barcodes corresponding to the number of cells aimed to be analyzed at the beginning of the library preparation (this value was 3,000 in our study). The software then checks the value of the UMI counts for the 99th percentile barcode (*i.e.* the barcode ranked last in the 1% of the barcodes with highest UMI counts; in our work, it would be the 30th barcode). Hence, the value "*m*" for the UMI counts of this barcode differs for each experiment (this value was 1,821 in our study). The UMI counts threshold to determine a reliable cell is then set at a tenth of *m* (in our case this number was set to

182.1). Hence, the Cell Ranger pipeline we used determined that barcodes presenting less than 182.1 UMI counts (so, lesser than 183) could not be qualified as reliable cells. (For more details, see https://kb.10xgenomics.com/hc/en-us/articles/115003480523-How-are-barcodes-classified-as-cell-associated-?). This process output a filtered gene expression matrix containing 2,045 barcodes identified as reliable cells. For further analyses, we exported this matrix to the Partek Flow software (Partek).

Single cell UMI counts were normalized and filtered to exclude genes expressed in less than 5% of the cells. Based on the calculation of the Davies-Bouldin index for optimal cell clustering, three clusters were generated. A t-distributed Stochastic Neighbor Embedding (t-SNE) algorithm was used to graphically visualize the sequenced cells. Sub-clustering has been processed by calculating the Davies-Bouldin index within each main cluster and picking the optimal number of sub-clusters greater than one. For building sub-cluster "Intermediary" ("Inter"), we manually included cells that were expressing genes associated with coat proteins that were contradictory to the main cluster they were initially assigned to. We also included cells that were still expressing *barp* while being assigned in a metacyclic cluster. The analyses for differential gene expression was generated using Partek Flow's Gene-Specific Analysis (GSA). For these analyses, gene expression values were assessed by the least-square means (LSM) of normalized UMI counts per cluster. To produce the heatmaps, LSM values have been adjusted to follow the standard normal distribution (for which the average= 0 and the variance= 1). Heatmaps were generated using R 3.5.1.

Differential transcript expression. RNA was extracted from BSF trypanosomes purified from three independently infected rat blood two weeks post infection initiation with BSF stabilates; from epimastigote and metacyclic parasite stages purified from three pools of infected tsetse SGs. Three groups of mice, each containing five animals, received $4x10^4$ SG purified parasites *via* needle inoculation ID in the ear. One group of mice that received SG purified parasites was sacrificed at each designated time, 36h, 96h and 7 days post parasite infection and total RNA was purified from each mouse blood sample using the Mouse RiboPure-Blood RNA isolation kit (Invitrogen, AM1951, California). Trypanosome gene-specific qRT-PCR primer sequences were identified using *Tbb*927 genome reference (*tritrypdb.org)* and the Primer-BLAST designer tool (https://www.ncbi.nlm.nih.gov/tools/primer-blast/) (primer sequences are shown in Table S1). All reactions were performed using a CFX96 Real-time system (Bio-Rad). Expression of targeted genes was normalized using the geometrical mean of the expression of three housekeeping genes (DNA Ligase I, AATP5, PM20). Housekeeping genes were selected for their stable expression based on RNAseq data obtained from the trypanosome located in the midgut, cardia and SG of tsetse flies (3). The multiple independent biological replicates of RNA extracts were used with 2 technical replicates per sample. Obtained Ct values and gene relative expression calculations are provided in Dataset S3.

Statistical significance was determined by a one-way ANOVA followed by a TukeyHSD posthoc test, using Prism 8.2 (GraphPad).

Gene Ontology (GO) Analysis. Specific GO terms enriched within the DE genes noted between different developmental stages were determined by Fisher's Exact Test using the Gene Ontology (GO) enrichment tool on the TriTrypDB webserver (http://tritrypdb.org/). The obtained GO terms were summarized by removing redundant terms using the REVIGO web-based software, setting up the allowed similarity at 0.5 (smaller list of GO terms).

Recombinant protein (rProtein) expression. Gene specific expression primers with unique restriction enzymes were designed to amplify the CDS's corresponding to the mature proteins without the signal peptide (Table S1). The expression insert was cloned into the pET-28a expression vector (Novagen, Wisconsin) and competent *Escherichia coli* BL21 cells were transformed. Expression of rSGE1 (Tb927.7.360, referred here as TbSGE1) was induced by addition of 1 mM IPTG and rSGE1 was purified using the His-Bind Purification Kit, according to manufacturer's instructions (catalog number 70239-3 Novagen, Wisconsin). Expression of rSGM1 (Tb927.7.6600, referred here as TbSGM1.7) was similarly induced by IPTG and purified using polypropylene columns (product number 29924, ThermoScientific, Illinois) with urea buffer (equilibration and binding buffer pH 8.0, wash buffer pH 6.3, elution buffer pH 4.5 with HCl). The purified proteins were analyzed by silver stain (Catalog number 1610449 Bio-Rad, California) on SDS-PAGE to ensure sufficient purity for immunization in mice.

Immunofluorescence analysis. For immunostaining, parasitized SG were severed into pieces in 100µl of PSG for 10 min at RT, and then fixed 30 min in a solution of 4% paraformaldehyde (PFA) and 0.2% gluteraldehyde. The solution was centrifuged for 5 min at 30g and the supernatant containing the trypanosomes was transferred to a fresh tube and centrifuged again for 10 min at 1000g. The pellet was resuspended in a fixing solution of 4% PFA at RT overnight. Fixed trypanosomes were then washed twice by centrifugation for 10 min at 1000g and resuspended in PBS. Slides were incubated with either rabbit anti-rSGE1 and mouse anti-rSGM1.7 sera or their respective pre-immune sera. Alexa Fluor 488-labeled goat anti-rabbit IgG (Invitrogen, California) and Alexa Fluor 594-labeled goat anti-mouse IgG were used as secondary antibodies. Lastly, slides were washed in PBST, rinsed in ddH_2O and the coverslip was mounted using VectorShield™ medium with DAPI (Vector Laboratories). Slides were visualized at either 400x or 1000x using a Zeiss Axio Imager M2 microscope and images were captured using an AxioCam Mrm and the AxioVision40 software (v4.8.2.0, Zeiss, Germany). Images were processed using the Fiji version of the ImageJ software. Only contrast and luminosity were adjusted on images.

Electron Microscopy. Metacyclic parasites were collected by incubating infected SGs in PSG buffer at RT for 45 min. The PSG buffer is composed of 6 parts of 0.1M Sodium Phosphate with 4 parts of water and glucose is added to 1% and pH adjusted to 8.0 prior to filter sterilization. Parasites were then fixed in 4% PFA and 0.2% gluteraldehyde for 30 min at RT. Parasites were then processed at the Yale Center for Cellular and Molecular Imaging (CCMI). Cells were pelleted by centrifugation at RT for 15 min at 500g and then fixed in 4%PFA overnight at RT. Pelleted cells were then rinsed in PBS and re-suspended in 10% gelatin. Trimmed blocks were placed in 2.3M sucrose overnight on a rotor at 4°C, then transferred to aluminum pins and frozen rapidly in liquid nitrogen. The frozen block was trimmed on a Leica Cryo-EMUC6 UltraCut and 65nm thick sections were collected, placed on a nickel formvar/carbon coated grid and floated in a dish of PBS ready for immunolabeling. The grids were placed section side down on drops of 0.1M ammonium chloride to quench untreated aldehyde groups, then blocked for nonspecific binding on 1% fish skin gelatin in PBS. For SGM1.7 labelling, grids were incubated either with pre-immune or anti-rSGM1.7 mouse sera at a dilution of 1:50. For SGE1 labelling, grids were incubated with either pre-immune or anti-rSGE1 rabbit sera at a dilution of 1:800. For SGM1.7 only, in the aim to improve labelling by bridging colloidal gold to the primary antibody, the grids were incubated with a rabbit anti-mouse IgG secondary antibody (JacksonImmuno) at a 1:200 dilution. For both SGM1.7 and SGE1, grids were then rinsed and incubated with 10nm colloidal gold-labelled protein-A (UtrechtUMC). Grids were then rinsed in PBS, lightly fixed using 1% glutaraldehyde for 5min, rinsed in PBS and finally transferred to a UA/methylcellulose drop, then collected and dried. Grids were viewed with a FEI Tencai Biotwin transmission electron microscope at 80Kv. Images were taken using a Morada CCD camera and the iTEM software (Olympus).

Antigenicity of rProteins in mice analyzed by ELISA. For immunizations, mice were given intraperitoneally (IP) 1ug purified rSGM1.7 or rSGE1 in PBS supplemented with a 1:1 ratio of Magic Mouse adjuvant (Creative Diagnostics, product number CDN-A001, New York). All mice received boosts after 2 and 4 weeks using the same protocol (described in Dataset S5). Two weeks post final boost, sera collected by eye bleed were analyzed by ELISA. 96-well flat-bottom Immuno Plate MaxiSorp certified plates (cat 439454 Nalge Nunc International, Rochester, NY) were coated with 100ng of each recProtein per well in 50µl coating buffer (0.1M Na_2CO_3, 0.1M $NHCO_3$, 1mM NaN_3, pH 9.6) for 2h at 24°C. The plates were incubated overnight at 4°C with serial dilutions of test and control sera (varying concentration from 1:500-1:1,562,500) in 50µl blocking buffer (0.1M PBS, pH 7.4, 0.05% Tween-20, 5% milk). Secondary antibody HRP-conjugated anti-mouse IgG (1:6000) (catalog no. W402B Promega, Madison Wisconsin), or HRP-conjugated anti-rabbit (1:12,000) diluted in blocking buffer was added for 1h at 24°C depending on the primary sera sample. The reaction was visualized

by the addition of 50µl chromogenic substrate (TMB, Catalog No 34021, Pierce Bioscience, Rockford Illinois) for 6-8 min. The reaction was terminated with 50µl H_2SO_4 and absorbance at 450nm was measured with reduction at 630 nm using ELISA plate reader (BioTek Synergy HT). Plates were washed five times with washing buffer (PBS, pH 7.4, containing 0.1% (v/v) Tween 20) after each step.

Metacyclic challenge of vaccinated mice. Vaccinated and age-matched control mice received, ID in the ear *via* needle injection, 500 parasites purified from infected SG which include epimastigotes, pre- and mature-metacyclic forms. Blood samples from all animals were collected via retro-orbital sampling daily on the first two days (days 4 and 5) and via tail bleeds on the next two days (days 6 and 7). For improving detection of parasite numbers, red blood cells were lysed. The RBC lysis solution is composed of 9 parts of stock 1 added to 1 part of stock 2 and the pH adjusted to 7.65 with 1M HCl. Stock 1 is 8.3 g ammonium chloride in 1,000 ml water, while stock 2 is 20.59 g Tris base in a total volume of 1,000 ml water adjusted to pH to 8.0 with 1M HCl. To lyse RBCs in parasitized blood, 1 part of blood is added to 9 parts (1:10 dilution) of the RBC lysis solution, mixed gently and allowed to stand for 15 min at RT before counting the parasites using a hemocytometer. Typically, we diluted 5 µl of parasitized blood with 45 µl of RBC lysis buffer and used 10µl per hemocytometer reading. When 5 or fewer parasites were detected in one hemocytometer field, we performed multiple hemocytometer counts from the total sample to get a more accurate estimation of the low parasitemia numbers. As the parasitemia increased, further dilutions were made for accurate counts. All experiments were performed with two groups of vaccinated mice with at least 5 individuals, independently. One group of 7 mice similarly vaccinated and 5 control mice (Experiment 13) were each challenged by a single infected tsetse fly bite intraperitoneally and mice were bled on days 3 to 7 and parasitemia determined microscopically. These data are provided in Dataset S5.

co-inoculation of metacyclic parasites with immune sera. Five immunized mice were cardiac-punctured, sera collected and IgG fraction purified using Nab protein A/G column and concentrated to 1 mg/ml (Thermo Scientific, product number 89958, Illinois). Two groups of BalbC female mice, (5 control and 5 rTbSGM1 vaccinated) were ID co-inoculated with 20µl rSGM1.7 IgG (corresponding to 20 µg) and 500 SG isolated parasites. Controls received the same number of SG isolated parasites inoculated with control mouse IgG. Mice were bled on days 4 to 7 and parasitemia determined microscopically as described above. The experiment was performed twice independently. Full data are provided in Dataset S5.

Supplementary Table

Table S1. List of primers used in this study.

Gene ID	Gene Name	Primer Function	Primer sense	Primer Sequence (5' to 3')	Reaction conditions
Tb927.7.6600	TbSGM1.7	rProtein expression	Forward	CGGGATCCGCAAGGGGCGTAACACTATA	98ºC-10sec, 60ºC-10sec, 72ºC-30sec x25 cycles
	TbSGM1.7	rProtein expression	Reverse	CCGCTCGAGTTCAGCAACAACAGCAGG	
Tb927.7.360	TbSGE1	rProtein expression	Forward	GGAATTCCATGACTCCATAATTGAGGAAGGGTCTC	98ºC-10sec, 55ºC-10sec, 72ºC-30sec x25 cycles
	TbSGE1	rProtein expression	Reverse	CCGCTCGAGCGGAAAATGTGCGGCAGCACTGTGAAAAAGTGCTGCAAGAAG	
Tb927.7.6600	TbSGM1.7	qPCR amplification	Forward	CACTTCCGGCAAACAGAACG	95ºC-15sec, 60ºC-30sec, 72ºC-30sec x40 cycles
	TbSGM1.7	qPCR amplification	Reverse	ACCTTTAGCGGTGTCAGTCG	
Tb927.6.400	Peptidase M20	qPCR amplification	Forward	GGGCGATGAGCTACGATCAA	95ºC-15sec, 60ºC-30sec, 72ºC-30sec x40 cycles
	Peptidase M20	qPCR amplification	Reverse	ACTGTGCATGCCTTCCTTCA	
Tb927.8.8290	Amino acid Transporter 5	qPCR amplification	Forward	TACGGGCAACCGACTTTTGA	95ºC-15sec, 60ºC-30sec, 72ºC-30sec x40 cycles
	Amino acid Transporter 5	qPCR amplification	Reverse	TAACGGCACAGGTGGAAACA	
Tb927.6.4780	DNA ligase I	qPCR amplication	Forward	AGCTTGAGGCCATCACCAAA	95ºC-15sec, 60ºC-30sec, 72ºC-30sec x40 cycles
	DNA ligase I	qPCR amplication	Reverse	TGAAACCACACATCCGGCTT	
Tb927.7.360	TbSGE1	qPCR amplification	Forward	GTAAGTGCGGCATGTCTC	95ºC-15sec, 55ºC-30sec, 72ºC-30sec x40 cycles
	TbSGE1	qPCR amplification	Reverse	CATCTTGGCAACCTTCTCTC	
mVSG ILTat 1.22	mVSG ILTat 1.22	qPCR amplification	Forward	AGGGCCAAGGTGTCATCAAG	95ºC-15sec, 60ºC-30sec, 72ºC-30sec x40 cycles
	mVSG ILTat 1.22	qPCR amplification	Reverse	GCTAATGGCTGCGTAGTTGC	
Tb927.7.6560	TbSGM1.3	qPCR amplification	Forward	ACGGAAAACGCTAACTCGGA	95ºC-15sec, 60ºC-30sec, 72ºC-30sec x40 cycles
	TbSGM1.3	qPCR amplification	Reverse	TCGCGCACGTCTTTCTTGTA	

Supplementary Figures

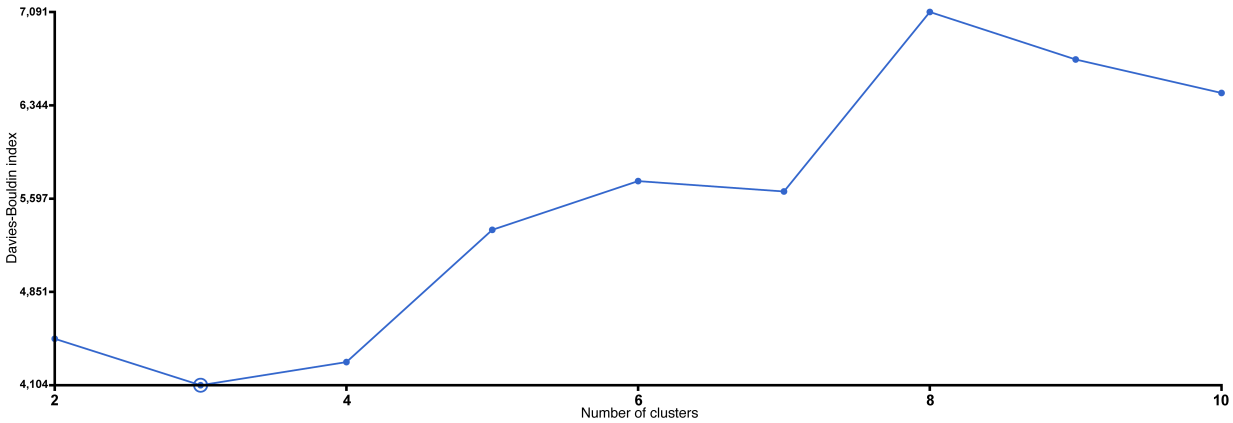

Fig. S1. Evaluation of optimal clustering using the Davies-Bouldin index.
Lower index indicates optimal clustering. Here, three clusters are optimal.

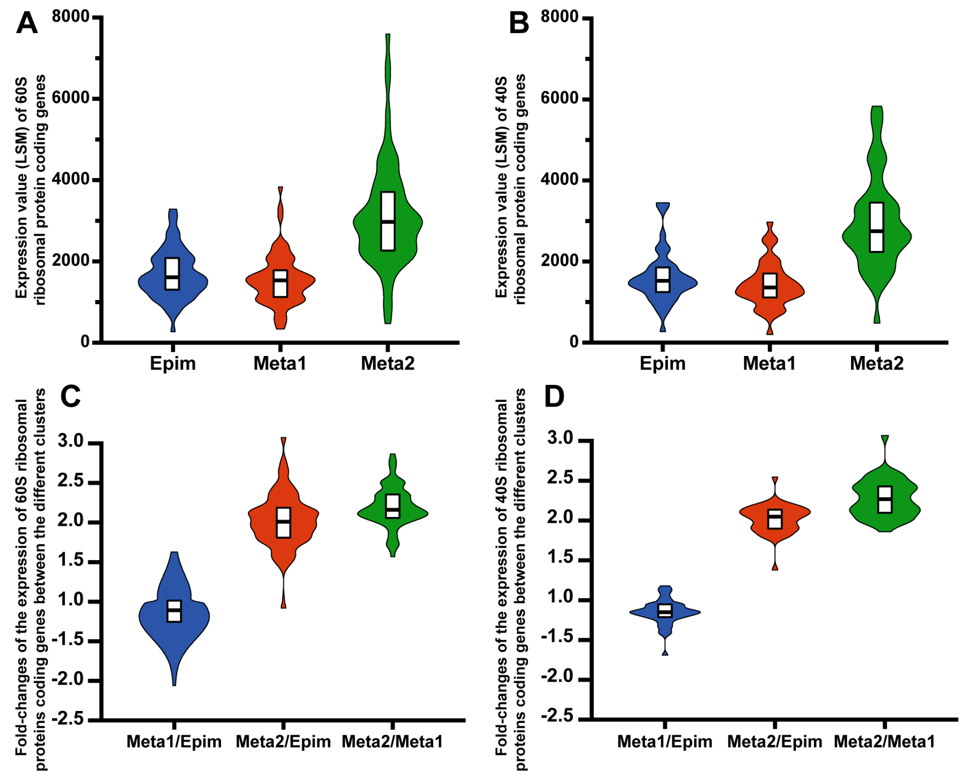

Fig. S2. Expression of Ribosomal proteins in each parasite cell cluster.

A and B. Violin plots of the expression value (LSM) of the genes coding for 60S-associated ribosomal proteins (A) and 40S-associated ribosomal proteins (B). Boxes represent the median and the first and third quartiles.

C and D. Violin plots of the fold-changes determined for each pairwise combination of the clusters for the genes coding for 60S-associated ribosomal proteins (C) and 40S-associated ribosomal proteins (D). Boxes represent the median and the first and third quartiles.

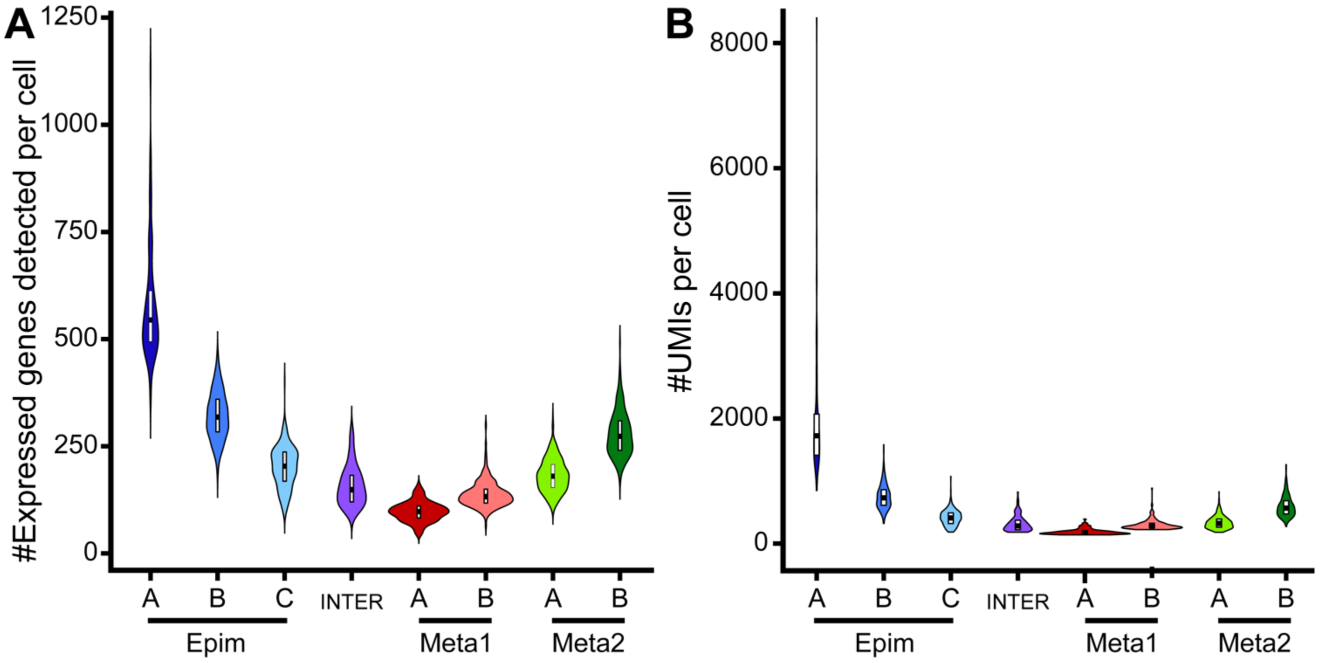

Fig. S3. Numbers of expressed genes and UMI counts per cell for the eight sub-clusters.
A. Violin plots of the number of expressed genes detected per cell for each sub-cluster.
B. Violin plots of the UMI counts per cell for each sub-cluster.
Boxes represent the median and the first and third quartiles.

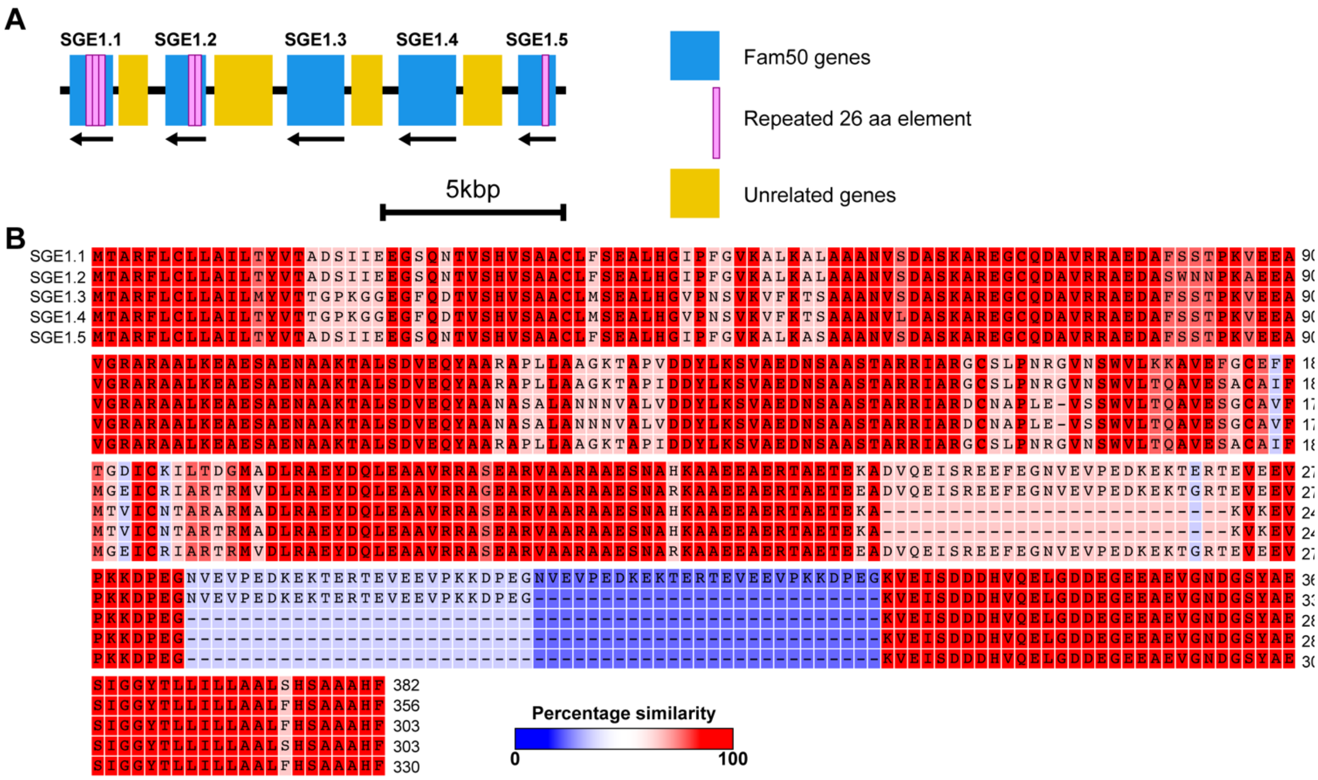

C

		1	2	3	4	5
SGE1.1	1		0.07	0.17	0.17	0.06
SGE1.2	2	86.65		0.18	0.18	0.02
SGE1.3	3	66.75	71.07		0.01	0.15
SGE1.4	4	67.02	71.07	98.68		0.15
SGE1.5	5	81.15	90.73	78.79	78.79	

Distance (upper triangle); Percentage similarity (lower triangle)

Fig. S4. Genomic characterization of the TbSGE1 family

A. The organization of the TbSGE1 family of genes localized on chromosome 7 between nucleotides 67,121 to 79,706. The genes are coded on the anti-sense strand. The yellow boxes denote unrelated genes, (*Tb927.7.430, Tb927.7.410, Tb927.7.390 and Tb927.7.370*) which reside between the different members of the *Tbsgm2* family. Pink bars denote the 26aa repeat sequence associated with each member of the family.

B. Amino acid alignment of the putative members of the TbSGE1 family (encoded by *Tbsge1.1-1.5*) generated by the alignment function of CLC (Qiagen). Percentage similarity between sequences is giving by the foreground color.

C. Percent identity between different members of the TbSGE1 family of proteins.

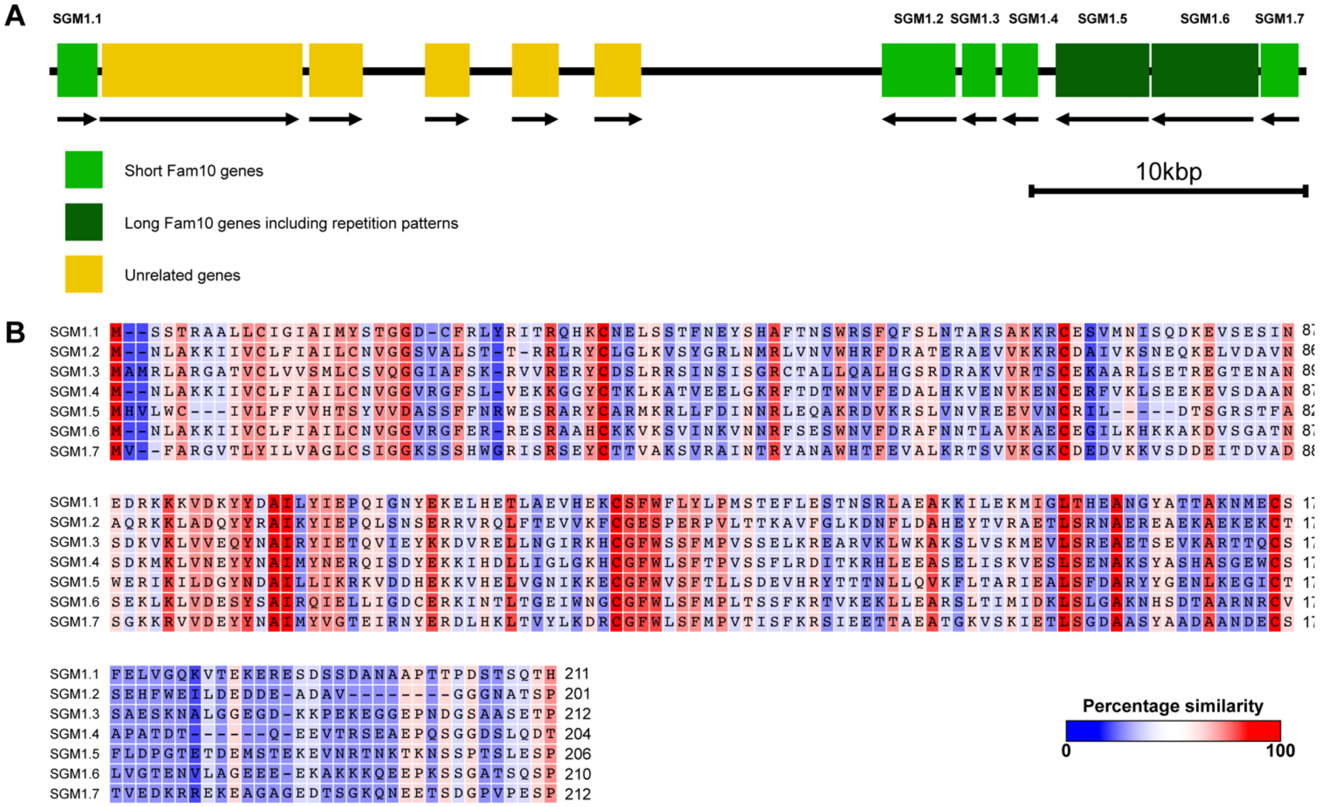

C

	1	2	3	4	5	6	7
SGM1.1 (1)		1.50	1.40	1.23	1.74	1.42	1.38
SGM1.2 (2)	23.11		1.35	1.04	1.59	1.10	1.35
SGM1.3 (3)	26.17	26.42		1.07	1.58	1.20	1.23
SGM1.4 (4)	29.72	34.29	34.43		1.43	0.85	1.13
SGM1.5 (5)	19.16	20.56	21.96	23.83		1.47	1.69
SGM1.6 (6)	25.94	33.33	31.60	42.38	23.83		1.18
SGM1.7 (7)	26.76	26.29	30.37	32.39	20.09	31.92	

Distance (upper triangle); Percentage similarity (lower triangle)

Fig. S5. Genomic characterization of the TbSGM1 family

Seven genes previously identified as Family 10 (Fam10) and shown to be specific for SG stages, were detected expressed in Meta1 to Meta2 sub-clusters. We named this family SGM1.

A. The chromosomal organization of Fam10 genes referred to as *sgm1.1* to *sgm1.7,* following their chromosomal localization.

B. Alignment of the protein coding sequences of the seven members of Fam10 family. Proteins have been truncated at the end due to the presence of a repetition sequence in SGM1.5 and SGM1.6.

C. The percent similarity between different members of Fam10 family on the non-repeated protein coding sequence (ranged from 19 to 42%, with *sgm1.5* and *sgm1.6* being the most divergent).

References

1. Moloo SK (1971) An artificial feeding technique for *Glossina*. *Parasitology* 63(3):507-512.
2. MacLeod ET, Maudlin I, Darby AC, & Welburn SC (2007) Antioxidants promote establishment of trypanosome infections in tsetse. *Parasitology* 134(Pt 6):827-831.
3. Savage AF*, et al.* (2016) Transcriptome Profiling of Trypanosoma brucei Development in the Tsetse Fly Vector Glossina morsitans. *PLoS One* 11(12):e0168877.
